# Genetic preservation of *SLC22A3* in the Admixed and Xhosa populations living in the Western Cape

**DOI:** 10.1007/s11033-023-08884-6

**Published:** 2023-11-04

**Authors:** Brendon Pearce, Clifford Jacobs, Mongi Benjeddou

**Affiliations:** 1 Genetics Department, Faculty of Agriscience, Stellenbosch University, Van Der Bijl Street, Stellenbosch, 7600 South Africa; 2 Department of Biotechnology, University of the Western Cape, Robert Sobukwe Road, Bellville, Cape Town, 7535 South Africa

**Keywords:** SLC22A3, Cape Admixed, Xhosa, Genotyping, Metformin

## Abstract

**Background:**

Amphiphilic solute facilitator organic cation transporters mediate the movement of various endogenous and exogenous organic cations, including crucial drugs like metformin, oxaliplatin, and lamivudine. These transporters are now seen as a potential explanation for inter-individual differences in drug effectiveness, contributing to 15–30% of such variability due to genetic factors.The aim of this study was to determine the baseline minor allele frequency distribution of 18 known coding SNPs in the *SLC22A3* gene of 278 Cape Admixed (130) and Xhosa (148) individuals residing in Cape Town, South Africa.

**Methods:**

A convenience sampling method was used for sample collection. DNA extraction and subsequent amplification of target sites was carried out according to standard established methodologies. All genotyping was performed using the SNaPshot™ mini-seuqencing platform.

**Results:**

This study found no genetic polymorphisms in the coding region of the *SLC22A3* gene of both the Xhosa and Cape Admixed individuals investigated.

**Conclusion:**

This study has shown that *SLC22A3* coding SNPs observed in other populations are absent in the sample of both Cape Admixed and Xhosa individuals studied. The lack of protein sequence variation was consistent with other studies and may reflect the significant physiological role of human organic cation transporter 3 in maintaining cellular and organismal homeostasis.

## Introduction

Organic cation transporters (OCTs) belong to the amphiphilic solute facilitator (ASF) family integral transmembrane proteins and are involved in various metabolic processes and detoxification Schömig, Spitzenberger [1]. These transporters are characterized by a specific organ and species-dependent expression and mediate the transport of organic cations (OCs) in an electrogenic and Na^+^-independent manner [2].

The human organic cation transporter 3 (hOCT3), also known as extraneuronal monoamine transporter (EMT), has a broad distribution and is found in various tissues, including the liver, heart, placenta, skeletal muscle, kidney, and brain [3, 4]. Moreover, hOCT3 is a polyspecific transporter that is involved in the cellular uptake and elimination of small OCs with different molecular structures. These OC substrates include endogenous bioamines, clinically important drugs and xenobiotics. Examples of substrates transported by hOCT3 include the antidiabetic metformin, the biogenic amines histamine, dopamine, and epinephrine, and the xenobiotics tetraethylammonium bromide (TEA) and the neurotoxin 1-methyl-4-pyridinium (MPP^+^) (Martel, 2003).

The gene encoding for hOCT3, *SLC22A3*, encodes a protein consisting of 556 amino acid residues and is located on chromosome 6 where it is clustered together with *SLC22A1 and SLC22A2* the genes coding for hOCT3’s paralogues hOCT1 and hOCT2, respectively [5, 6]. Seminal work by, Chen et al. (2013) demonstrated that genetic polymorphisms in the proximal promoter region of *SLC22A3* alter the transcription rate of the gene and may be associated with altered expression levels of hOCT3 in the liver [7]. In addition, they also showed that hypermethylation of the CpG island in the proximal promoter region is the probable mechanism accounting for decreased expression of hOCT3 in prostate cancer.

The ubiquitously expressed hOCT3 has also increasingly been recognized as an important transporter of anti-cancer drugs. For example, a study by Yokoo et al. (2008) investigated whether hOCT3 was significantly involved in oxaliplatin-induced cytotoxicity and accumulation of platinum in colorectal cancer [8]. It was concluded that hOCT3-mediated uptake of oxaliplatin into cancer cells was indeed important for its toxicity, and that hOCT3 may be a marker for cancer chemotherapy. In another study, Shnitsar et al. (2009) found that renal cell carcinoma (RCC) cell lines, usually chemoresistant, expressing hOCT3 increases chemosensitivity to the antineoplastics, melphalan, irinotecan, and vincristine [9]. In a study by Li et al. (2012) found that hOCT3 also partially contributed to the sensitivity of human cervical adenocarcinoma cells to cisplatin cytotoxicity [10]. Most recently, Hsu et al. (2017) showed that upregulation of *SLC22A3* expression improved cisplatin uptake in vitro in squamous cell carcinoma cells, demonstrating a possible mechanism by which patient survival may be improved [11].

The hOCT3 gene, *SLC22A3*, was also identified as an important risk locus for prostate cancer, and was markedly under-expressed in aggressive prostate cancers [12]. This study also revealed that hypermethylation of the *SLC22A3* promoter region in prostate cancer was one of the important mechanisms for the reduced expression of this transporter. Furthermore, a study by Mohelnikova-Duchonova et al. (2013) found a significant upregulation of *SLC22A3* in pancreatic ductal adenocarcinoma (PDAC) tumours compared to non-neoplastic tissues [13]. In addition to cancer, the *SLC22A3-LPAL2-LPA* gene cluster was also previously identified in a genome-wide association (GWAS) haplotype study as a risk locus for coronary artery disease (CAD) [14].

The biguanide antidiabetic drug metformin is usually the first-line therapeutic used in the treatment of type-2 diabetes [15, 16]. The action of metformin appears to be related to its activation (phosphorylation) of the energy sensor AMP-activated kinase (AMPK), which results in suppression glucagon-stimulated glucose production and enhancement of glucose uptake in muscle and hepatic cells [17, 18]. Previous studies have shown that OCTs, the hOCT3 paralogues hOCT1 and hOCT2, together with multidrug and toxin extrusion (*MATE*) genes (play a critical role in the disposition response and that genetic variants of these transporters are associated with variation in pharmacokinetic and anti-diabetic action of the drug [19–23]. Subsequently, a study by Chen et al. (2010) has suggested that in addition to hOCT1, hOCT2 and *MATE1*, hOCT3 should be considered an important mechanism for metformin uptake in muscle cell types and that variation in this transporter may modulate the response to metformin [24]. Further developments in pharmacokinetic research have suggested that while the current evidence in pharmacokinetic variability linked to recognized OCTs/MATEs genotypes is generally limited, it may still hold significance in terms of tissue-specific effects and for drugs with a narrow therapeutic index [25].

To bridge the gap in pharmacogenetic mapping in African populations, especially those residing in southern Africa, this study prioritized the genotyping of 18 known variable sites in the coding region of the *SLC22A3* gene in the Cape Admixed and Xhosa populations living in the Cape Town, South Africa.

## Materials and methods

### Compliance with ethics guidelines

Samples were obtained from the participants with informed consent. This study was approved by the Senate Research Ethics Committee of the University of the Western Cape, South Africa. In addition, all procedures followed were in accordance with the ethical standards of the responsible committee on human experimentation (institutional and national) and with the Helsinki Declaration of 1975, as revised in 2000 (5).

### Subjects

Biological samples were collected, via convenience sampling from a healthy population, in the form of buccal swabs from 130 to 148 unrelated healthy volunteers from the Cape Admixed and Xhosa populations, respectively. Ethnicity of volunteers was determined by self-report.

### DNA extraction and SNP selection

Isolation of genomic DNA from buccal swab samples was carried out using a standard salt-lysis protocol and stored frozen at -20 °C until the time of genotyping [26]. A total of 18 *SLC22A3* coding SNPs were selected for this study. SNPs were selected from the literature and the Ensembl database (http://www.ensebl.org) [27]. Variants N162I; A169T; R212H; M248V; G269E; R293C; R310C; S337F; R348W; I381T; V388M; R403H; R407H; I431K; and R490Q were included in this study based on predicted effect on function, using the SIFT (Sorting Intolerant From Tolerant) program [28–30]. To our knowledge no population data exist in the public domain for these variants.

### Primer design

Multiplex PCR primers for the amplification of all 11 *SLC22A3* exons and flanking regions were designed using Primer3 software (www.genome.wi.mit.edu/cgi-bin/primer/primer3) and are listed in Table 1. To test for possible non-specific amplification, primers were aligned with the NCBI sequence databases using Basic Local Alignment Search Tool (www.ncbi.nlm.nih.gov/blast/blast-cgi). Two SNaPshot™ Multiplex systems were specifically designed for the study, successfully optimized and used for genotyping. The single base extension primer sets for multiplex 1 and 2 are listed in Tables 2 and 3.

**Table 1:** Multiplex PCR primers for the generation of *SLC22A3* amplicons used in SNaPshot™ genotyping

Location	Forward primer (5´to 3´)	Reverse Primer (5ʹto 3ʹ)	Amplified region (NC_000006.12)	Amplicon length (bp)
Exon 2	TGCATTCTGGCATGTCTCCATGTGT	ACCGGGAACAGCCTCAGACCT	160,397,935–160,398,311	377
Exon 3	GTTTAAGGTGAGCTCTTTTCCTGT	TTGGCTCCCAAAGTAAGGTGG	160,407,004–160,407,404	401
Exon 4	CTGCAAGTGTGGAAGCCTCCGT	GCTGGGCAGCGTGATGGCTA	160,408,607–160,408,898	292
Exon 5	TGCAGGAATAATCTGTATTTCAGGG	ACTGAAAATGATTTCCCAGATGTT	160,410,569–160,411,034	466
Exon 6 & 7	TGAAAGCCCCTAGTCACTTCAG	TGGAGTGACATCACGAAAGACT	160,436,664–160,437,340	677
Exon 8	CTTCAGACTGGAGGCCACTAAGCA	ACGCTGGTCTACAGAGTTACTTAG	160,442,659–160,442,921	263
Exon 9	GGATAACACCCTCCACCCAC	ACTGAATTGGCTCTCAAAACTG	160,443,405–160,443,934	530
Exon 10	TGTTTCCCTGTGATGCAGGA	TGCTTCTCTCTTCACAACCACAT	160,447,401–160,448,051	651
Exon 11	TGATCCTGGAGACAGATATTGTTGT	GTCAGAGACCACAGGGAACA	160,450,844–160,451,347	504

**Table 2:** *SLC22A3* gene multiplex 1 single base extension primers for SNaPshot™ genotyping assay of selected SNPs.

NCBI (dbSNP)	Amino Acid Change	Nucleotide change	Nucleotide sequence (5′ → 3′)	Position Accession number (NC_000006.12)	Primer length (bp)	polyGACT tail
rs183669984	R310C	C > T	AAAGGAGATAAAGCATTACAGATCCTGAGA	160,410,799	30	0
rs137958808	M370I	G > T	AAGCGCAGTGGTGTATCAAGGACTTGTCAT	160,437,033	35	5
rs199688797	R212H	G > A	CACCAAACTTCCCTGTGTTTGTGATCTTCC	160,407,142	40	10
rs150004342	A169T	G > A	ACCTGTCTGCTGCATAGCCTAAGGTGAATG	160,398,054	45	15
rs142228053	R293C	C > T	TTCTTTGCCAGGGTGGTCCCTGAGTCTCCC	160,410,748	50	20
rs149424049	I431K	T > A	AATGTAGCCACTGTGGTCCTCAACCATGCT	160,442,764	55	25
rs147863404	G269E	G > A	AAGCTGGGCAGCGTGATGGCTAACTGGATT	160,408,870	60	30
rs8187725	T400I	C > T	AAGGGGAGGCGTCGTCCAAGGCGCTCAATG	160,437,122	65	35
rs149101094	M248V	A > G	CAAAGGAGGATTGTGGGAATCGTGATTCAA	160,408,806	70	40
rs141104413	S337F	C > T	CTGTTACAGATGAGGAAGTTAGTAATCCAT	160,436,814	75	45

**Table 3:** *SLC22A3* gene multiplex 2 single base extension primers for SNaPshot™ genotyping of selected SNPs.

NCBI (dbSNP)	Amino Acid Change	Nucleotide change	Nucleotide sequence (5′ → 3′)	Position Accession number (NC_000006.12)	Primer length (bp)	polyGACT tail
rs139266499	N162I	A > T	GGATGCTGGACCTCACCCAAGCCATCCTGA	161,081,878	35	5
rs145328121	R348W	A > T	TTTTTAGATCTGGTGAGAACTCCCCAAATG	160,436,846	45	15
rs187750009	I381T	T > C	GCCTGGGAATTATAGGGGGCAACCTCTATA	160,437,065	50	20
rs189883656	V388M	G > A	AGATCAAGAGAGCTCCTGGCAGTTCCACCA	160,437,085	55	25
rs200478210	R403H	G > A	GAGCTCTCTTGATCTTACTAACCATTGAGC	160,437,131	60	30
rs145082363	R407H	G > A	GCCACTATATTGCTTGCCGCAAAGGGGAGG	160,437,143	65	35
rs12212246	A439V	C > T	CAGGAATAGCATGGTTGAGGACCACAGTGG	160,442,788	70	40
rs144856002	R490Q	G > A	AGAGGTAGTTCTAGCCACACGGCTGCTAGC	160,443,701	75	45

### Multiplex PCR

The PCR reactions were performed in a 20 *µ*l volume, containing 20–50 ng of genomic DNA, 1 x Qiagen multiplex PCR master mix (Qiagen, Courtaboeuf, France) and 0.2 *µ*M of each primer. Cycling consisted of an initial 15 min activation step for HotStar Taq polymerase at 95 °C, followed by a total of 35 cycles using the following conditions: 94 °C denaturation for 30 s, primer annealing at 60 °C for 90 s, and primer extension at 72 °C for 30 s, and 15 min of final extension at 72 °C and a 4 °C holding step. PCR products were purified to remove excess primers and un-incorporated dNTPs using an Exonucleas (Exo)/Shrimp Alkaline Phosphatase (SAP) protocol. The entire 20 *µ*l of PCR products were incubated with 0.5 *µ*l of Exo1 and 1 *µ*l of SAP for 30 min at 37 °C followed by 15 min at 80 °C for enzyme inactivation. PCR quality and yield were checked using NanoDrop.

### Multiplex mini-sequencing reactions

Multiplex mini-sequencing was performed in a 10 *µ*l reaction volume using 3 *µ*l of a 1/10 dilution of purified PCR products, 0.1–0.2 *µ*M of primers, and 5 *µ*l of SNaPshot™ ready reaction mix (Applied Biosystems). Sequence cycling consisted of 25 cycles of denaturation at 96 °C for 10 s, primer annealing at 50 °C for 5 s, and primer extension at 60 °C for 30 s. Post-extension treatment was done by adding 1 U of SAP to the 10 µl reaction volume and incubation at 37 °C for 30 min followed by 15 min at 80 °C to deactivate the enzyme.

### Electrophoresis of the mini-sequencing products

The purified mini-sequencing products (1 *µ*l) were mixed with 8.7 *µ*l of HiDi™ formamide and 0.3 *µ*l of GeneScan-120 Liz size standard (Applied Biosystems) and denatured at 95 °C for 5 min. The fluorescently labelled fragments were separated on 36 cm-long capillaries in POP4 polymer on an ABI Prism 3500 Genetic Analyzer (Applied Biosystems). Data analyses were performed using GeneMapper® IDX Software Version 1.2.

## Results and discussion

The population studied consisted of 130 Cape Admixed and 148 Xhosa individuals between the ages of 18 and 72 years. There were 196 (71%) female and 84 (29%) male participants.

In this study we have developed two SNaPshot™ multiplex assays for genotyping 18 known nonsynonymous coding SNPs in the *SLC22A3* gene. The genotype and allele frequencies of the 18 *SLC22A3* gene SNPs investigated in our subjects are summarized in Table 4. All 18 coding SNPs genotyped in this study were monomorphic in the both the Cape Admixed and Xhosa populations.

**Table 4:** Genotype and allele frequencies of the OCT3 *(SLC22A3)* gene SNPs in 278 healthy individuals

Amino Acid Substitution	dbSNP ID	Observed Genotype Frequency				Allele Frequency			
		Genotype	Cape Admixed	Xhosa	95% CI	Allele	%	95% CI	HWE (P)
**N162I**	**rs139266499**	**AA**	100.0	100.0	96.9–100.0	A	100.0	98.4–100.0	
		**AT**	0.0	0.0	0.0–1.3	T	0.0	0.0–1.6	
		**TT**	0.0	0.0	0.0–1.3				
**A169T**	**rs150004342**	**GG**	100.0	100.0	96.9–100.0	G	100.0	98.4–100.0	
		**GA**	0.0	0.0	0.0–1.3	A	0.0	0.0–1.6	
		**AA**	0.0	0.0	0.0–1.3				
**R212H**	**rs199688797**	**GG**	100.0	100.0	96.9–100.0	G	100.0	98.4–100.0	
		**GA**	0.0	0.0	0.0–1.3	A	0.0	0.0–1.6	
		**AA**	0.0	0.0	0.0–1.3				
**M248V**	**rs149101094**	**AA**	100.0	100.0	96.9–100.0	A	100.0	98.4–100.0	
		**AG**	0.0	0.0	0.0–1.3	G	0.0	0.0 -1.6	
		**GG**	0.0	0.0	0.0–1.3				
**G269E**	**rs147863404**	**GG**	100.0	100.0	96.9–100.0	G	100.0	98.4–100.0	
		**GA**	0.0	0.0	0.0–1.3	A	0.0	0.0 -1.6	
		**AA**	0.0	0.0	0.0–1.3				
**R293C**	**rs142228053**	**CC**	100.0	100.0	96.9–100.0	C	100.0	98.4–100.0	
		**CT**	0.0	0.0	0.0–1.3	T	0.0	0.0–1.6	
		**TT**	0.0	0.0	0.0–1.3				
**R310C**	**rs183669984**	**CC**	100.0	100.0	96.9–100.0	C	100.0	98.4–100.0	
		**CT**	0.0	0.0	0.0–1.3	T	0.0	0.0–1.6	
		**TT**	0.0	0.0	0.0–1.3				
**S337F**	**rs141104413**	**CC**	100.0	100.0	96.9–100.0	C	100.0	98.4–100.0	
		**CT**	0.0	0.0	0.0–1.3	T	0.0	0.0 -1.6	
		**TT**	0.0	0.0	0.0–1.3				
**R348W**	**rs145328121**	**AA**	100.0	100.0	96.9–100.0	A	100.0	98.4–100.0	
		**AT**	0.0	0.0	0.0–1.3	T	0.0	0.0 -1.6	
		**TT**	0.0	0.0	0.0–1.3				
**M370I**	**rs137958808**	**GG**	100.0	100.0	96.9–100.0	G	100.0	98.4–100.0	
		**GT**	0.0	0.0	0.0–1.3	T	0.0	0.0–1.6	
		**TT**	0.0	0.0	0.0–1.3				
**I381T**	**rs187750009**	**TT**	100.0	100.0	96.9–100.0	T	100.0	98.4–100.0	
		**TC**	0.0	0.0	0.0–1.3	C	0.0	0.0–1.6	
		**CC**	0.0	0.0	0.0–1.3				
**V388M**	**rs189883656**	**GG**	100.0	100.0	96.9–100.0	G	100.0	98.4–100.0	
		**GA**	0.0	0.0	0.0–1.3	A	0.0	0.0–1.6	
		**AA**	0.0	0.0	0.0–1.3				
**T400I**	**rs8187725**	**CC**	100.0	100.0	96.9–100.0	C	100.0	98.4–100.0	
		**CT**	0.0	0.0	0.0–1.3	T	0.0	0.0–1.6	
		**TT**	0.0	0.0	0.0–1.3				
**R403H**	**rs200478210**	**GG**	100.0	100.0	96.9–100.0	G	100.0	98.4–100.0	
		**GA**	0.0	0.0	0.0–1.3	A	0.0	0.0–1.6	
		**AA**	0.0	0.0	0.0–1.3				
**R407H**	**rs145082363**	**GG**	100.0	100.0	96.9–100.0	G	100.0	98.4–100.0	
		**GA**	0.0	0.0	0.0–1.3	A	0.0	0.0–1.6	
		**AA**	0.0	0.0	0.0–1.3				
**A439V**	**rs12212246**	**CC**	100.0	100.0	96.9–100.0	C	100.0	98.4–100.0	
		**CT**	0.0	0.0	0.0–1.3	T	0.0	0.0–1.6	
		**TT**	0.0	0.0	0.0–1.3				
**I431K**	**rs149424049**	**TT**	100.0	100.0	96.9–100.0	T	100.0	98.4–100.0	
		**TA**	0.0	0.0	0.0–1.3	A	0.0	0.0–1.6	
		**AA**	0.0	0.0	0.0–1.3				
**R490Q**	**rs144856002**	**GG**	100.0	100.0	96.9–100.0	G	100.0	98.4–100.0	
		**GA**	0.0	0.0	0.0–1.3	A	0.0	0.0–1.6	
		**AA**	0.0	0.0	0.0–1.3				

Over the last number of years hOCT3 has increasingly being recognized as an anti-diabetic and anti-cancer drug transporter [8, 24, 31]. Several reports provide evidence of the increased interest in the role of hOCT3 in neurotransmission and maintenance of homeostasis in the central nervous system (CNS) as a result of its recognized ability to translocate monoamines [32–34]. In addition, hOCT3 is also drawing interest as a potential target in the treatment of selected neuropsychiatric disorders.

In the current study we genotyped 18 known SNPs in the *SLC22A3* gene of ~ 140 Cape Admixed and Xhosa individuals residing in the Cape Town metropolitan area, South Africa. We observed no genetic variation for the 18 noncoding SNPs genotyped in the investigated population. However, this lack of genetic variation in the coding region of *SLC22A3* is not a unique situation and has also been observed in other populations (Table 5) [35, 36]. Moreover,, this high degree of genetic preservation and lack of protein sequence variation may reflect the crucial physiological role hOCT3 plays in maintaining homeostasis [37, 38].

**Table 5:** Minor allele frequency comparison of the 18 selected SNPs between representative global populations

dbSNP ID	Minor Allele	MAF (%) by Population								
		Cape Admixed	Xhosa	Luhuya (Kenya)	Esan (Nigeria)	British European	American European	Admixed Latin American	East Asian	South Asian
**rs139266499**	**T**	**0**	**0**	―	―	―	―	―	―	―
**rs150004342**	**A**	**0**	**0**	**0**	**0**	**0**	**0**	**0**	**0**	**0**
**rs199688797**	**A**	**0**	**0**	**0**	**0**	**0**	**0**	**0**	**0**	**0**
**rs149101094**	**G**	**0**	**0**	**0**	**0**	**0**	**0**	**0**	**0**	**0**
**rs147863404**	**A**	**0**	**0**	**0**	**0**	**0**	**0**	**0**	**0**	**0**
**rs142228053**	**T**	**0**	**0**	**0**	**0**	**0**	**0**	**0**	**0**	**0**
**rs183669984**	**T**	**0**	**0**	**0**	**0**	**0**	**0**	**0**	**0**	**0**
**rs141104413**	**T**	**0**	**0**	**0**	**0**	**0**	**0**	**0**	**0**	**0**
**rs145328121**	**T**	**0**	**0**	**0**	**0**	**0**	**0**	**0.0001**	**0**	**0**
**rs137958808**	**T**	**0**	**0**	**0**	**0**	**0**	**0**	**0**	**0**	**0**
**rs187750009**	**C**	**0**	**0**	**0**	**0**	**0**	**0**	**0**	**0**	**0**
**rs189883656**	**A**	**0**	**0**	**0**	**0**	**0**	**0**	**0**	**0**	**0**
**rs8187725**	**T**	**0**	**0**	**0**	**0**	**0**	**0**	**0.0001**	**0**	**0**
**rs200478210**	**A**	**0**	**0**	**0**	**0**	**0**	**0**	**0**	**0.001**	**0**
**rs145082363**	**A**	**0**	**0**	**0**	**0**	**0**	**0**	**0.0001**	**0.001**	**0**
**rs12212246**	**T**	**0**	**0**	**0**	**0**	**0**	**0**	**0**	**0**	**0**
**rs149424049**	**A**	**0**	**0**	**0**	**0**	**0**	**0**	**0**	**0**	**0**
**rs144856002**	**A**	**0**	**0**	**0**	**0**	**0**	**0**	**0**	**0**	**0**

The ubiquitously expressed hOCT3 has not only been implicated in the transport of anti-cancer drugs, but also as a biomarker for cancer pathogenesis [39]. For example, in colorectal cancers hOCT together with its paralogues, hOCT1 and hOCT2, have been shown to be determinants of oxaliplatin cytotoxicity [8, 39–41]. Moreover, *SLC22A3* expression in renal cell carcinoma cell lines enhances the sensitivity of these cell lines towards the chemotherapeutic agents melphalan, irinotecan, and vincristine [9]. Cui et al. (2011) identified the *SLC22A3* SNP rs7758229 as a risk locus for distal colon cancer in an Asian population. More recently, however, Yusuf et al. (2021) confirmed these findings through polygenic modelling, associating rs7758229 with colorectal cancer risk [42, 43]. In addition, a study by Grisanzio et al. (2012) showed that *SLC22A3* is inversely correlated with prostate cancer progression, with markedly decreased expression in aggressive prostate cancers [44].

Metformin is a biguanide anti-diabetic drug and is widely used as a first-line therapeutic in the treatment of type-2 diabetes. Earlier studies have shown that metformin is transported by OCT1 and OCT2, and that genetic polymorphisms of these transporters affect the pharmacokinetic and therapeutic effect of the drug [19–23]. Studies have also implicated human *MATEs* and hOCT3 in metformin absorption, disposition, and pharmacological action [20, 24, 25]. Chen et al. (2010) found that the OCT3 variant T400I significantly reduced metformin uptake by the transporter [24]. Structural modelling suggested that this variant may be located in the pore lining of the TMHs, where it plays a critical role in substrate translocation. The T400I variant is a rare variant that has a low allele frequency and was not observed in the individuals that participated in this study. Given the prevalence of type-2 diabetes in South Africa and the widespread use of metformin as a therapeutic, the distribution of this variant in the indigenous African populations require further investigation. The effect of this variant in vivo on metformin pharmacokinetics and efficacy has not been demonstrated yet,but should be assessed if the T400I variant is identified in any of the indigenous African populations.

Ideally a larger sample size and complete sequencing of the *SLC22A3* gene would provide a more complete picture of the spectrum of genetic variation within this gene for these populations. In addition, several SNPs in the proximal promoter region had been associated with altered expression of the *SLC22A3* gene previously, however, the current approach of genotyping coding SNPs only, excluded these variants from being assayed. Furthermore, although medical research has primarily focused on protein-coding variants, this picture has changed with advances in the systematic annotation of functional non-coding elements [45]. However, the genotyping strategy adopted in this study have excluded the typing of non-coding SNPs, which could be useful when performing linkage disequilibrium analysis or extracting information about disease association.

## Conclusions

To our knowledge this study represents the first of its kind to investigate the baseline allele and genotype frequency distributions of known genetic polymorphisms within the *SLC22A3* gene of the Cape Admixed and Xhosa populations. This study has shown that *SLC22A3* coding SNPs observed in other populations are absent in the sample of individuals studied. The lack of protein sequence variation, ascribed to selective pressures that act at the *SLC22A3* locus, was found to be consistent with other studies and may reflect the significant physiological role of hOCT3 in maintaining cellular and organismal homeostasis.

## Data Availability

All data is available from the authors upon reasonable request.

## List of abbreviations

AMPK: AMP-activated kinase
ASF: Amphiphilic solute facilitator
CAD: Coronary artery disease
CNS: Central nervous system
DDIs: Drug-drug interactions
EMT: Extraneuronal monoamine transporter
GWAS: Genome-wide association
hOCT3: Human organic cation transporter 3
MATE: Multidrug and toxin extrusion
MDCK: Madin-Darby canine kidney
MPP^+^: 1-Methyl-4-pyridinium
OCTs: Organic cation transportes
TEA: Tetraethylammonium bromide
